# Supplementary material for: Individual variability in functional connectivity architecture of the mouse brain
Source: Commun Biol. 2020 Dec 4;3:738. doi: 10.1038/s42003-020-01472-5 (PMC7718219; doi:10.1038/s42003-020-01472-5)
Supplement: Supplementary file 1 — Supplementary Information [file 42003_2020_1472_MOESM1_ESM.pdf]

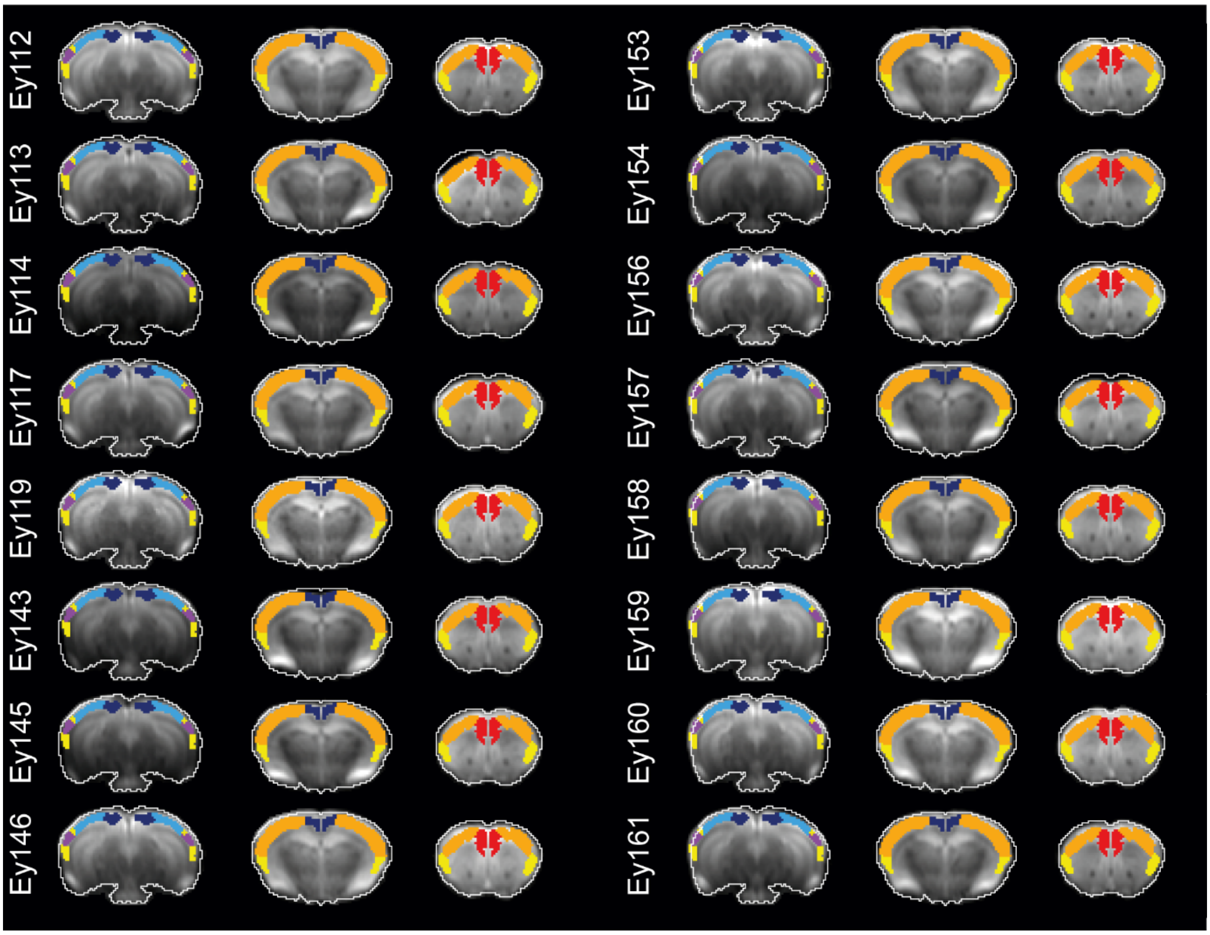

Figure S1. Comparison of registration quality between mice. Average SE-EPI (fMRI) coronal slices from all sessions of each mouse with overlaid cortical labels. The white frames represent the masked group average image as shown in **Figure 1**.

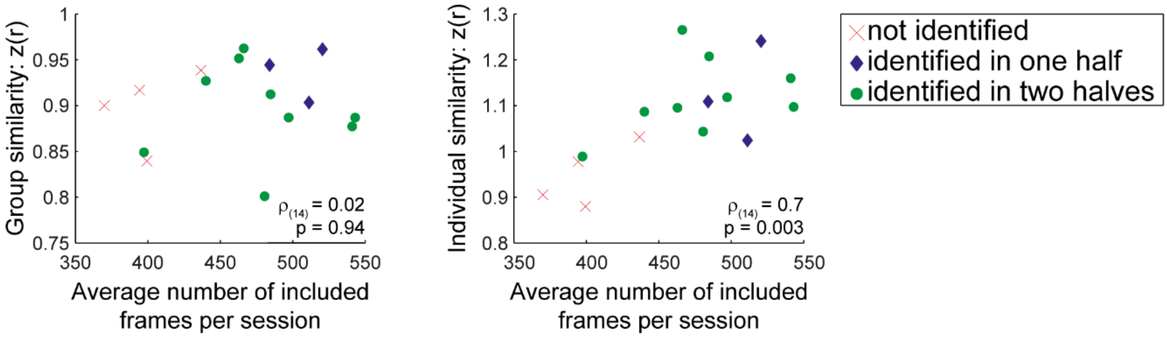

Figure S2. The effect of motion on estimation of individual variation. Sessions with increased head movement have lower number of included frames after motion scrubbing. The average number of included frames was compared to group similarity (*left*) and individual similarity (*right*), as well as to the identifiability of different mice in the two halves of data. Overall, the results show that the average number of included frames is significantly correlated with individual, but not group, similarity, and that mice with increased scrubbing are less identifiable. Therefore, we conclude that motion reduces the estimation of individual variation rather than contributes to it.

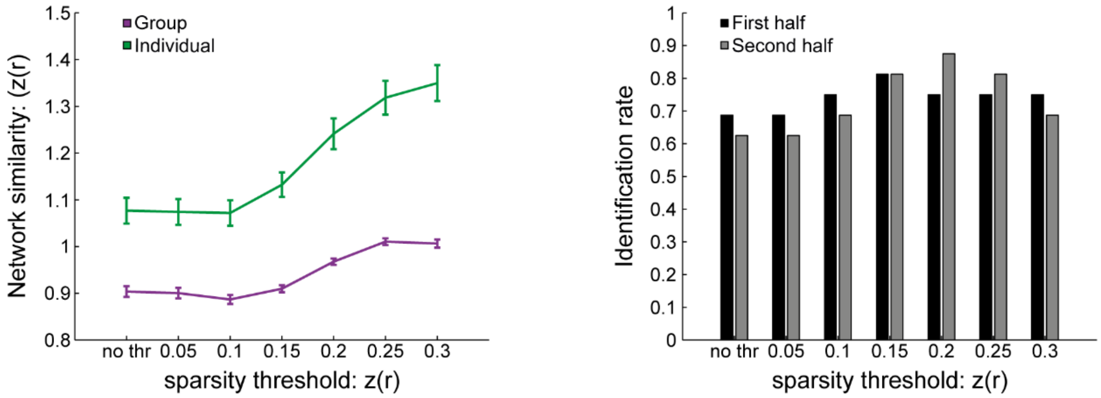

Figure S3. The effect of connectome sparsity on estimation of individual variation. *Left,* to validate that the observed individual variation is not caused by spurious correlation, we used correlation thresholds from the group average connectivity matrix to define sparser connectomes and examine their group and individual network similarity; error bars represent the standard error of the mean (n = 16 mice). Repeated-measures ANOVA with Individuality and Sparsity Threshold as within mouse factor revealed significant effect of both factors (Individuality: $F_{(1, 15)} = 64.92$, $P < 0.001$, $\varepsilon_{H\text{-}F} = 1$, $\eta^2 = 0.812$; Sparsity Threshold: $F_{(6, 90)} = 11.65$, $P < 0.001$, $\varepsilon_{H\text{-}F} = 0.224$, $\eta2 = 0.882$), as well as a significant interaction between them ($F_{(6, 90)} = 39.53$, $P < 0.001$, $\varepsilon_{H\text{-}F} = 0.206$, $\eta^2 = 0.725$), suggesting that sparsity improves the estimation of individual variation. *Right*, examining the identifiability of individual connectomes at different thresholds reveals better performance than the original analysis with peak identification rate of over 0.8 in medium thresholds.

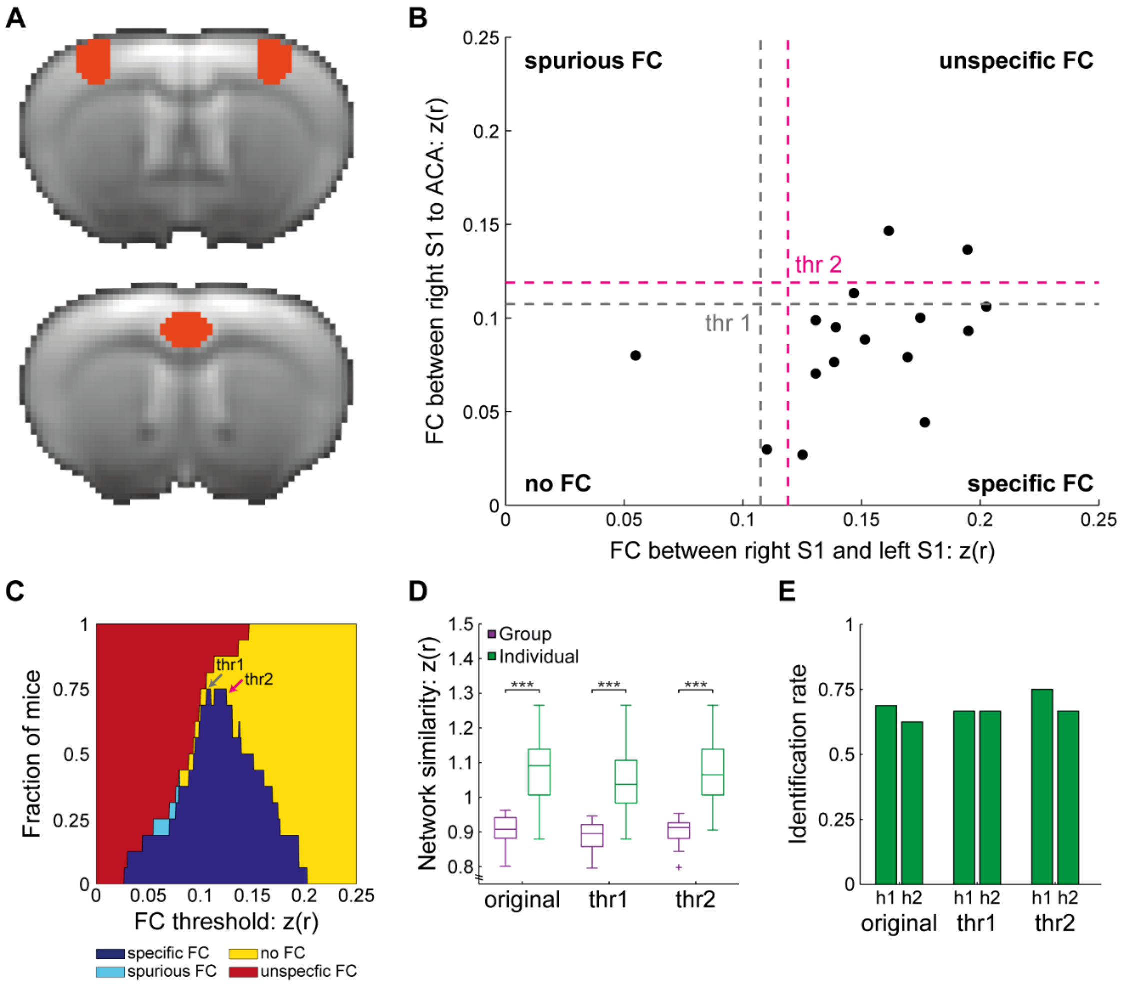

Figure S4. The effect of functional connectivity specificity on estimation of individual variation. (A) Location of seed regions in primary somatosensory cortex (S1, *top*) and anterior cingulate cortex (ACA, *bottom*). (B) Comparison between individual-level functional connectivity (FC) of the right S1 to either left S1 (specific ROI) or ACA (non-specific ROI) reveals bias toward the specific ROI in 15 out of 16 mice (paired student *t*-test : $t_{(15)} = 6.6$, $P < 0.001$); z(r) values were averaged across the six sessions of each mouse. Two z(r) thresholds (thr1 in *grey*, thr2 in *magenta*) were used for the classification of each mouse connectivity profile as "specific FC", "unspecific FC", "spurious FC" or "no FC" as described in Grandjean et al.[33]; the results show specific FC in 12 out of 16 mice. (C) The distribution of FC specificity as a function of z(r) threshold reveals two peaks (thr1, thr2), which were used for the definition of two subgroups in which individual variation was estimated to validate that the original results are not driven by mice with non-specific FC. (D) Comparison of group and individual network similarity between the original group (n = 16 mice) and the two subgroups (n = 12 mice in each subgroup) reveals higher similarity in individuals in all cases (***$P < 0.001$, paired student *t*-test: original: $t_{(15)} = 7.32$, $P < 0.001$; thr1: $t_{(11)} = 5.6$, $P < 0.001$; thr2 $t_{(11)} = 6.1$, $P < 0.001$); boxplots represent the median (center line), interquartile range (box limits); 1.5 × interquartile range (whiskers) and outlier (crosses). (E) Comparison of identification rates between the original group and the two subgroups reveal similar values, suggesting that the original results are not driven by mice with non-specific FC. h1, first half of data; h2, second half.

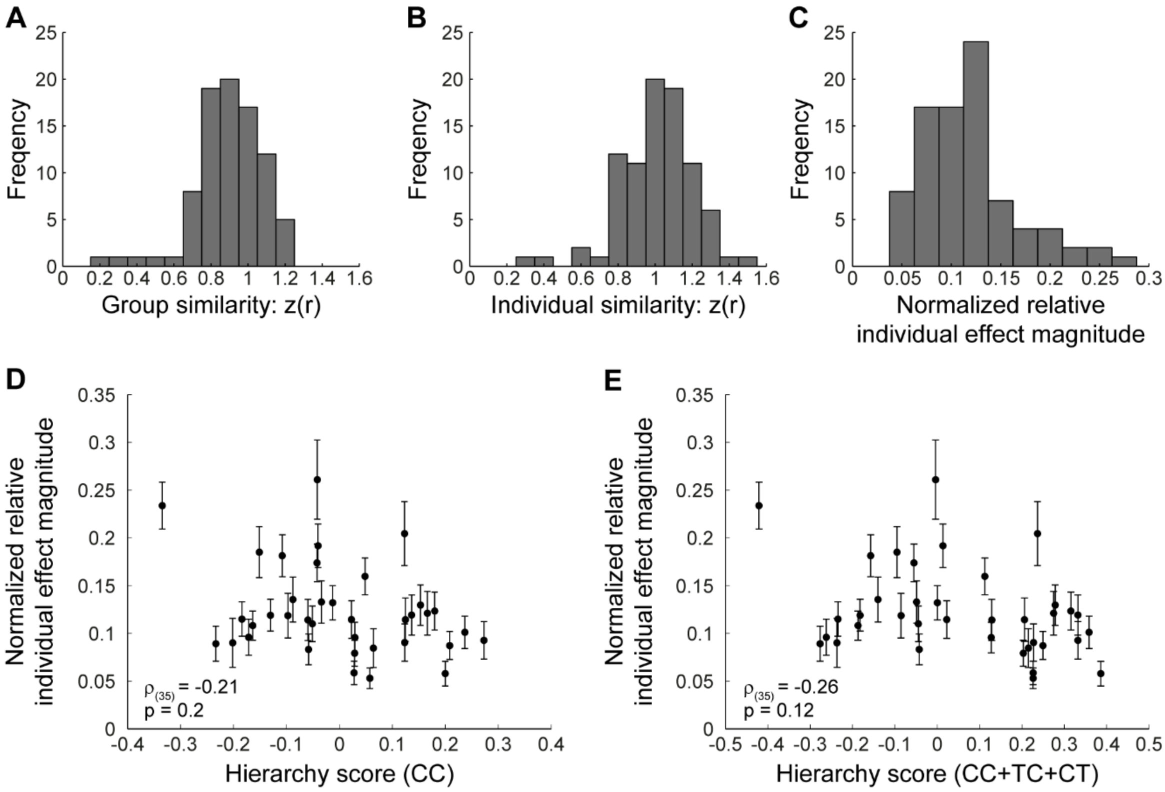

Figure S5. Comparison between parcel-level individual variation and anatomical hierarchy. Distributions of average group similarity (A), individual similarity (B) and normalized relative individual effect magnitude (C) over 86 cortical parcels. Comparison between normalized relative individual effect magnitude and anatomical hierarchy taken from the work of Harris et al.[34]. Two types of hierarchy scores were taken based on corticocortical connections (D) or corticocortical, thalamocortical and corticothalamic connections (E). In both cases non-significant negative correlations were observed. Error bars represent that standard error of the mean (n = 16 mice). Note that values from left and right cortical regions were averaged. CC, corticocortical; TC, thalamocortical; CT, corticothalamic.
