# Supplementary material for: Individual variability in functional connectivity architecture of the mouse brain
Source: Commun Biol. 2020 Dec 4;3:738. doi: 10.1038/s42003-020-01472-5 (PMC7718219; doi:10.1038/s42003-020-01472-5)
Supplement: Supplementary file 2 — Description of Additional Supplementary Files [file 42003_2020_1472_MOESM2_ESM.pdf]

Description of Additional Supplementary Files

File Name: Supplementary Data 1
Description: Parcel-level group and individual similarities

File Name: Supplementary Data 2
Description: Data underlying the article's figures.
